# Patterns of Antimicrobials Prescribed to Patients Admitted to a Tertiary Care Hospital: A Prescription Quality Audit

**DOI:** 10.7759/cureus.15896

**Published:** 2021-06-24

**Authors:** Aduragbenro D Adedapo, Onyinye O Akunne

**Affiliations:** 1 Pharmacology and Therapeutics/Pharmacoepidemiology, University of Ibadan, Ibadan, NGA; 2 Internal Medicine/Clinical Pharmacology, University College Hospital, Ibadan, NGA; 3 Pharmacology and Therapeutics, University of Ibadan, Ibadan, NGA

**Keywords:** antibiotics, in-patients, rational prescribing, atcc, ddd

## Abstract

Introduction

Rational use of antimicrobial agents is necessary to prevent the emergence of drug resistance. This study aims to assess the prescription pattern of antibiotics using the Anatomic Therapeutic Chemical Classification (ATCC)/Defined Daily Dose (DDD) metrics in real-world practice.

Methods

A retrospective audit of antibiotics prescribed to patients admitted to a tertiary hospital over 20 months. The demographics and clinical information of patients were collected. The ATCC/DDD system was used to classify antibiotics. The DDD per 100 bed-days was calculated and the quality of prescription, including generic and parenteral formulation use, was evaluated.

Results

Nine-hundred ninety-four prescriptions were analyzed. The average number of antibiotics prescribed was 2±1. Only 23% of the patients had confirmed cases of bacterial infection. Imidazole derivatives (J01X) were the most prescribed antibiotics (68.8 DDDs per 100 bed-days) followed by cephalosporins (45.0 DDDs), beta-lactams (35.3 DDDs), fluoroquinolones (30.9 DDDs), and macrolides/lincosamides (14.4 DDDs). Sulphonamides/trimethoprim (4.7 DDD), aminoglycosides (0.8 DDD), penicillin (0.3 DDD), and carbapenems (0.1 DDD) were the least prescribed. Metronidazole was the most prescribed drug (34.2%). Generic names and parenteral formulations were used in 55% and 72% of antibiotics prescribed.

Conclusion

The continued low generics prescribing calls for interventions to be put in place to improve prescribing quality. Parenteral formulation prescribing encountered was very high, though this may not be unexpected in in-patients, it is vital to curtail the use of parenteral formulations so as to minimize the risk of infection.

Irrational antibiotics prescription remains a serious concern in Nigeria. Drug utilization research using the ATCC/DDD metric is helpful in monitoring trends of drug use over time. This will help improve antibiotics stewardship and promote the rational use of antibiotics.

## Introduction

Rational antimicrobial use is necessary to prevent bacterial resistance and improve clinical outcomes while reducing the cost of treatment. The utilization of antibiotics needs to be evaluated frequently to promote effective prescribing. These frequent audits will expose the type, magnitude, and reason for irrational prescribing if present. The magnitude of overuse or irrational use needs to be known to adequately reduce antimicrobial use, particularly where there is no ongoing bacterial infection [1]. Patients admitted to hospitals are frequently exposed to antibiotics, most times without an ongoing infection [2]. In resource-limited settings like Nigeria, laboratory tests are not always ordered or test results are not readily available or not carried out [3].

The Anatomic Therapeutic Chemical Classification (ATCC)/Defined Daily Dose (DDD) system is effective in monitoring the rational and irrational use of drugs [4]. Examples of ATCC include tetracyclines (J01A), penicillin (J01C), beta-lactams (J01C), cephalosporins (J01D), carbapenems (J01D), sulphonamides/trimethoprim (J01E), macrolides/lincosamides (J01F), aminoglycosides (J01G), fluoroquinolones (J01M), and imidazole derivatives (J01X). It can be useful in studying national trends over time [5]. Knowledge of antimicrobial consumption patterns will aid in understanding the reason for irrational drug use. Studies have shown the DDD per 100 bed-days to be between 44.6 and 86.2 DDDs [6-8].

Studies on in-patient antibiotics prescription in Nigeria show that less than 60% of patients received antibiotics for a bacterial infection and the most frequently prescribed antibiotic drug classes are Imidazole derivatives, cephalosporins, fluoroquinolones, and B-lactams [9-10]. A study from a large standard community pharmacy showed similar findings [11]. Low generic prescription of antibiotics and high use of parenteral formulations have also been observed. Ilyasu et al. (2015) found generic prescription of antibiotics to be about 60% and parenteral formulations use to be about 80% [9]. Generic prescription is necessary to reduce out-of-pocket expenditure, especially in Nigeria where the cost of medication is borne by the patients. The use of parenteral formulations is costly and increases the risk of infection [1], therefore, it should be reduced where possible.

The prescription of antibiotics in Nigeria was recently evaluated [12], however, in this point prevalence survey, consumption per DDD was not evaluated. The hypothesis of this study stated as the null hypothesis (H0) is that there is no irrational use of antimicrobials among patients admitted into the medical wards. The research question is what is the pattern of antimicrobials use among medical in-patients?

Objective 1

To assess the prescription of antibiotics using the Anatomic Therapeutic Chemical Classification (ATCC)/Defined Daily Dose (DDD) metrics. To determine what constituted 90% (DU90) of antimicrobials use among the patients.

Objective 2

To compare study findings with the prescription patterns of antibiotics in other regions in Nigeria. This study, therefore, assessed the pattern of antibiotic prescription and quantified the amount of antibiotics prescribed to in-patients using the ATCC/DDD system. Drug utilization 90% (DU90%), the total drugs making up 90% of the total antibiotics prescribed, was determined. The pattern of antibiotics prescription is further compared to two other studies in Nigeria to understand the shift in the trend of antibiotics prescription.

## Materials and methods

This was a retrospective evaluation of a larger prospective study that was carried out in the six medical wards of the University College Hospital, Ibadan, Nigeria, between 2012 and 2013, for which ethical approval and written informed consent were obtained [13]. In-patients receiving an antibiotic for systemic treatment of bacterial infection were evaluated. Patients prescribed topical antibiotics and those on anti-tuberculosis regimens were excluded from this study.

Patients’ demographics and clinical information were collected from patients’ medical records. The information included the names and number of antibiotics prescribed to patients, dosage, delivery mode and frequency of administration, the reason for admission, duration of hospital stay, and patient outcome. The ATCC/DDD system was used for the classification of antibiotics and the measurement of doses prescribed. Antibiotics were classified as imidazole derivatives (J01X), penicillin (J01C), beta-lactams (J01C), cephalosporins (J01D), carbapenems (J01D), sulphonamides/trimethoprim (J01E), macrolides/lincosamides (J01F), aminoglycosides (J01G), fluoroquinolones (J01M), and tetracyclines (J01A). The DDD/100 bed-days was calculated using the WHO Applications of the ATCC/DDD methodology [14] as: 

DDDs for each antibiotic were obtained from the WHO ATC DDD index. A patient day was defined as an overnight stay in the hospital.

The drug utilization 90% (DU90%), the total drugs making up 90% of the total antibiotics prescribed, was measured. The generic prescription and the number of parenteral formulations prescribed were also evaluated.

Two studies on antibiotics prescription carried out in in-patients [9-10] were identified for comparison. The first study was carried out in the same time period as our study (2012) [9]; the second study was carried out in 2019 [10]. The presence or absence of a shift in antibiotics prescription over the time period was assessed.

Data analysis was performed using SPSS version 23 (IBM Corp., Armonk, NY) and Microsoft Excel (Microsoft Corporation, Redmond, WA). Frequencies, percentages, and means were used to present findings.

## Results

Of the 1280 prescriptions of inpatients reviewed during the study period, 994 prescriptions had complete dosage information. Antibiotics were prescribed to 462 patients. Most patients were hospitalized for less than one month with a mean hospital stay of 13±14 days. Males accounted for 55% of the study population, the mean age was 50±19. The average number of antibiotics prescribed was 2±1. The most frequent reason for admission was cardiovascular diseases (13.4%). Only about 23% of the patients had confirmed cases of bacterial infection. Most admitted patients recovered (65%) (Table 1).

**Table 1:** Demographic and clinical variables of in-patients admitted treated with antibiotics

Variable	Number (462)	% (100)
*Age (years)*		
<20	3	7.8
21 – 30	57	12.3
31 – 40	84	18.2
41 – 50	59	12.8
51 – 60	81	17.5
61 – 70	75	16.2
≥70	70	15.2
Duration of hospital stay (Months)		
<1	426	92.2
1 – 3	35	7.6
>3	1	0.2
No. of antibiotics prescribed		
1	125	27.1
2	228	49.4
3	84	18.2
4	19	4.1
5	5	1.1
6	1	0.2
Disease		
Cardiovascular diseases	62	13.4
Viral diseases	39	8.4
Tuberculosis	38	8.2
Cerebrovascular diseases	37	8
Diabetes mellitus	36	7.8
Sepsis	36	7.8
Other bacterial Infections	30	6.5
Renal diseases	30	6.5
Pulmonary diseases	26	5.6
Ulcers	15	3.2
Cancer	14	3
Gastrointestinal diseases	14	3
Hepatic diseases	12	2.6
Other viral diseases	10	2.2
Asthma	4	0.9
Malaria	4	0.9
Others	55	11.9
Outcome of Treatment		
Recovered	300	65
Dead	99	21.3
Discharged against medical advice	20	4.3
Transferred	43	9.4

The total DDD/100 bed-days prescribed during the study period was 200. Imidazole derivatives (J01X) were the most prescribed antibiotics; 68.8 DDDs per 100 bed-days were prescribed (Table 2), and metronidazole accounted for 100% of the antibiotics in this drug class. The second-most prescribed antibiotic class was cephalosporins (J01D). A total of 45.0 DDDs of cephalosporins were prescribed. Ceftriaxone accounted for 85% of cephalosporins prescribed; cefuroxime, cefixime, and ceftazidime made up 11%, 3%, and 1% of the prescription, respectively. The DDD per 100-bed-days of beta-lactams (J01C) prescribed was 35.3 DDDs; amoxicillin and clavulanic acid made up 98% of the prescription; flucloxacillin made up 2% of the prescription. Fluoroquinolones (J01M) were prescribed in 201 patients with 30.9 DDDs per 100 bed-days. Ciprofloxacin made up 94% of fluoroquinolones prescribed, levofloxacin made up 5%, and ofloxacin made up 1%. The DDDs per 100 bed-days of macrolides/lincosamides (J01F) prescribed was 14.4 DDDs (azithromycin, 89%; clarithromycin, 8%; clindamycin, 3%), sulphonamides/trimethoprim (J01E) was 4.7 DDD (sulfamethoxazole/trimethoprim, 100%); penicillin (J01C) was 0.3 DDD (amoxicillin, 100%), carbapenems (J01D; meropenem, 100%), and aminoglycosides (J01G; amikacin, 40%; gentamycin, 40%; neomycin, 20%) were 0.1 and 0.8 DDD, respectively (Table 2).

**Table 2:** Distribution of daily defined doses of antibiotic drug classes *DDDs per 100 bed days; DDDs: daily defined doses

Antibiotic class (ATCC Code)	Number of prescriptions	Number of DDDs*
Imidazole derivatives (J01X)	340	68.8
Metronidazole (J01XD01)	340	68.8
Cephalosporins (J01D)	203	45.0
Ceftriaxone (J01DD04)	172	36.2
Cefuroxime (J01DD02)	23	6.5
Cefixime (J01DD08)	6	2.2
Ceftazidime (J01DD02)	2	0.2
Beta-lactams (J01C)	169	35.3
Amoxicillin/Clavulanic acid (J01CR02)	165	34.7
Flucloxacillin (J01CF05)	4	0.7
Fluoroquinolones (J01M)	201	30.9
Ciprofloxacin (J01MA02)	188	26.2
Levofloxacin (J01MA12)	11	3.8
Ofloxacin (J01MA01)	2	0.9
Macrolides/Lincosamides (J01F)	37	14.4
Azithromycin (J01FA10)	33	13.1
Clarithromycin (J01FA09)	3	0.9
Clindamycin (J01FF01)	1	0.4
Sulphonamides/Trimethoprim (J01E)	37	4.7
Sulfamethoxazole/Trimethoprim (J01EE01)	37	4.7
Penicillin (J01C)	1	0.3
Amoxicillin (J01CA04)	1	0.3
Carbapenems (J01D)	1	0.1
Meropenem (J01DH02)	1	0.1
Aminoglycosides (J01G)	5	0.8
Amikacin (J01GB06)	2	0.4
Neomycin (J01GB05)	1	0.2
Gentamycin (J01GB03)	2	0.2
Total	994	200

The antibiotics making up 90% of the total antibiotics prescribed (DU 90%) included metronidazole (34.2%), ciprofloxacin (18.9%), ceftriaxone (17.3%), amoxicillin/clavulanic acid (16.6%), and sulfamethoxazole/trimethoprim (3.7%) (Table 3).

**Table 3:** Drug utilization 90% of antibiotics prescribed to in-patients

ATCC Code	Antibiotic prescribed	Number of antibiotics prescribed	% antibiotics prescribed
J01XD01	Metronidazole	340	34.2
J01MA02	Ciprofloxacin	188	18.9
J01DD04	Ceftriaxone	172	17.3
J01CR02	Amoxicillin/Clavulanic acid	165	16.6
J01EE01	Sulfamethoxazole/Trimethoprim	37	3.7
		902	90.7

**Figure 1:**
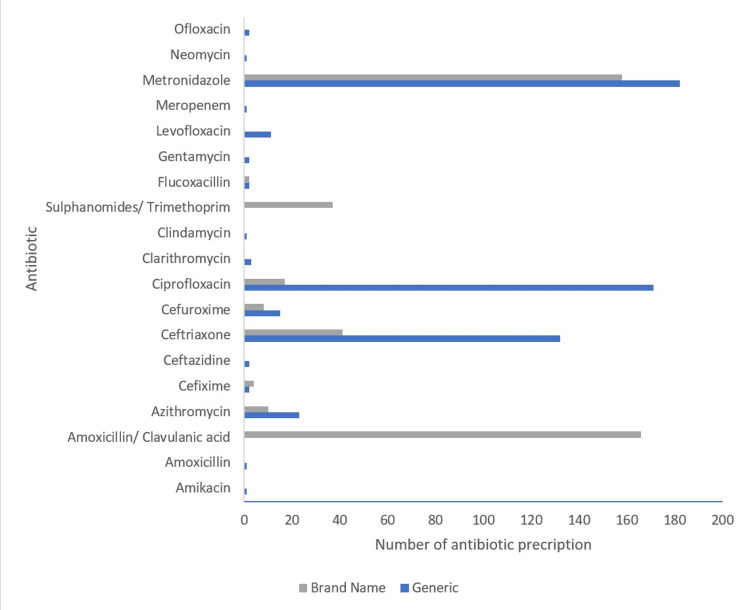
Distribution of antibiotics prescribed by generic or brand names

Generic names were used in 55% of antibiotics prescribed (Figure 1).

**Figure 2:**
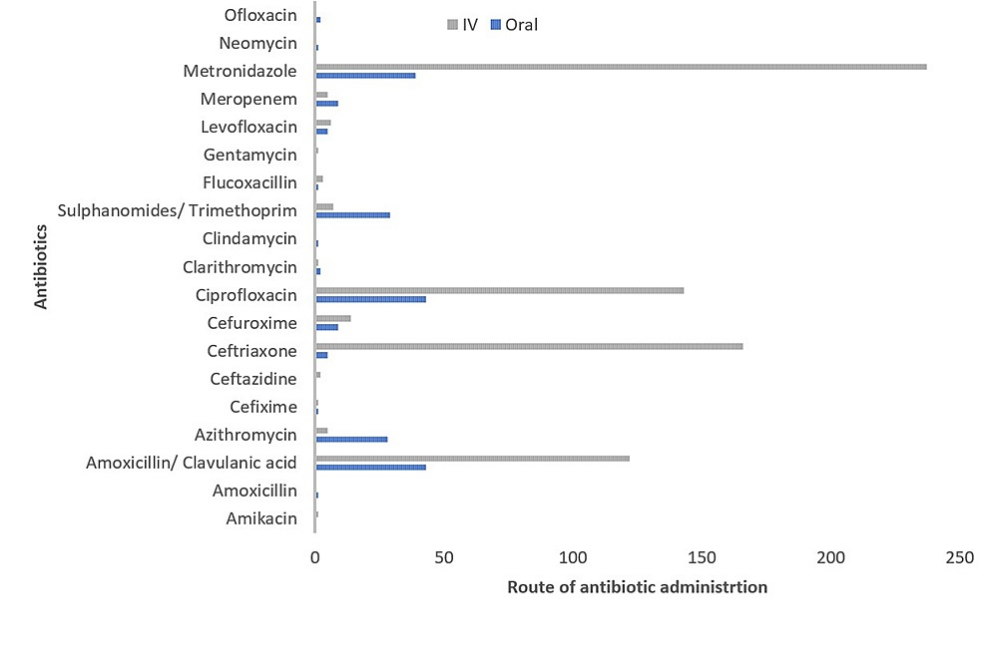
Distribution of prescribed antibiotic routes of administration

Intravenous administration was the most common route of antibiotic delivery accounting for 72% of prescribed antibiotic administration (Figure 2).

**Figure 3:**
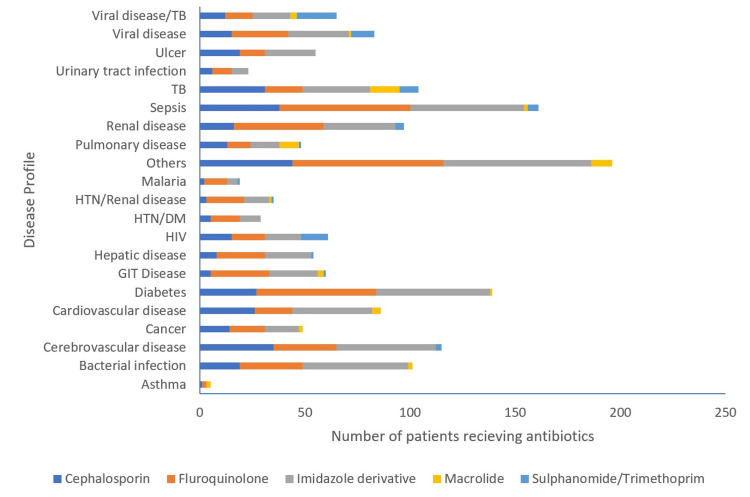
Disease/antibiotic prescription profile of patients admitted to the wards

Patients with sepsis and diabetes mellitus most often received antibiotics, infrequently others with malaria and viral diseases as well as cardiovascular diseases also received antibiotics (Figure 3). 

## Discussion

Our study shows that bacterial infection was confirmed in less than a quarter of the patients. Findings from another study carried out in Nigeria show that antibiotics are frequently prescribed to in-patients without confirmed bacterial infection [3]. This is also true in other developing countries. A study carried out in India showed frequent prescribing of antimicrobials to inpatients without bacterial infections presenting with viral fever, malaria, and cardiovascular disease [2]. Prescription of an antibiotic without confirmed or suspected infection has been shown to be caused by the prescribers’ anxiety of missing an infection [15]. We compared our study findings with data from a study carried out in the northern region of Nigeria in 2012 in inpatients and found a marked difference in the class of antibiotics prescribed. Cephalosporins were the most prescribed antibiotic drug class (40.4%); ceftriaxone was also the most prevalent cephalosporin prescribed. It accounted for 98% of the total cephalosporins prescribed. In both our study and theirs, the parenteral route was the commonly prescribed route of administration (72%, 81%, respectively) and the generic prescription was average (55%, 62%, respectively) (Table 4). Another study carried out in Northern Nigeria in 2019 showed findings consistent with our study findings. Marked differences were observed in the prescription of sulphonamides/trimethoprim, aminoglycosides, and penicillins. They were prescribed to 0.9%, 8.5%, and 8.5% of the patients, respectively, whereas in our study, they were prescribed to 3.7%, 0.5%, and 0.1% of the patients, respectively (Table 4). There was an observed reduction in the prescription of injections in the recent study, although the percentage of injections prescribed was still above average. The average number of antibiotics per patient encounter was 2 in all three studies (Table 4).

**Table 4:** Comparison of antibiotic drug prescription in 2012/2013 and 2019 *Mean ± SD; **Median (IQR, interquartile range)

Amoxicillin/Clavulanic acid	165 (16.6)	42 (12.6)	40 (12.5)
Sulfamethoxazole/Trimethoprim	37 (3.7)	-	4 (1.2)
Azithromycin	33 (3.3)	23(6.9)	6 (1.9)
Cefuroxime	23 (2.3)	2 (0.6)	17 (5.3)
Levofloxacin	11 (1.1)	2 (0.6)	6 (1.9)
Cefixime	6 (0.6)	-	22 (6.9)
Flucloxacillin	4 (0.4)	4 (1.2)	-
Clarithromycin	3 (0.3)	29 (8.7)	-
Amikacin	2 (0.2)	-	-
Ceftazidime	2 (0.2)	1 (0.3)	9 (2.8)
Gentamycin	2 (0.2)	1 (0.3)	38 (11.8)
Ofloxacin	2 (0.2)	1 (0.3)	-
Amoxicillin	1 (0.1)	1 (0.3)	18 (5.6)
Clindamycin	1 (0.1)	-	14 (4.4)
Meropenem	1 (0.1)	-	1 (0.3)
Neomycin	1 (0.1)	4 (1.2)	-

Irrational prescribing of antibiotics has been shown to promote bacterial resistance to treatment [16]. It is very important to have an audit system in place to curb excessive antimicrobial prescriptions. Antimicrobial resistance is a global epidemic and an imminent time bomb; it is important to prevent resistance by methods such as rational prescription through institutionalized prescription audits or drug utilization studies [17]. Sadly, many developing countries, including Nigeria, are yet to implement such measures, hence a rise in bacterial resistance.

The total number of DDDs per 100 patient days prescribed was 200 DDD. This is much higher than the DDD of 44.6 - 86.2 for the period 2012-2013 reported in other studies [6-8]. The ATCC/DDD metric is very useful in monitoring trends in drug use. For antibiotics, this could be important in improving antibiotic stewardship. Changes in the volume of DDDs, particularly where large changes were observed, serve as a red flag warranting further studies to improve antibiotics use. For example, a study from Iran reported a jump in antibiotics consumption from 33.6 DDDs per 1000 inhabitants per day (DID) to 60 DID from 2000 to 2016. It was also noted that Iran’s consumption of antibiotics was triple that of other OECD countries [18]. Evaluation of the reason behind this jump will help in the formulation of guidelines and other necessary interventions to improve antimicrobial use.

In our study, the DDD of metronidazole was the highest. In other studies, cephalosporins had the highest DDD (27.7-41.3 DDDs) [6-8]. Only one study reported the DDD of metronidazole (3 DDD). This clearly shows a difference in the prescribing pattern of antibiotics in our region and other regions.

Metronidazole, ciprofloxacin, ceftriaxone, amoxicillin/clavulanic acid, and sulfamethoxazole/trimethoprim made up about 90%of prescribed antibiotics. This distribution is similar to the two other studies [9-10] although sulfamethoxazole/trimethoprim was not commonly prescribed in the other studies. This may be a consequence of the most stocked antibiotics and availability. A study, though on children, assessed the availability of 27 antibiotics in 21 countries based on AWaRe (access, watch, reserve) antibiotics categories of the World Health Organization’s 2019 list of essential medicines found co-trimoxazole and metronidazole were most widely available, being in stock at 89.5% (interquartile range, IQR: 11.6%) and 87.1% (IQR: 15.9%) of health facilities, respectively. Of the 22,699 children observed, 60.1% (13,638) were prescribed antibiotics (mostly co-trimoxazole or amoxicillin) [19]. More than 50% of the medication prescribed were broad-spectrum antibiotics. In the case of suspected bacterial infection, broad-spectrum antibiotics can be used as empiric treatment. Appropriate empirical antibiotic treatment has been associated with a reduced medical cost and a better clinical outcome in patients with microbial infections [20]. Correct antimicrobial treatment should be implemented once laboratory results confirming the causal pathogen are received.

Prescription of antibiotics by their generic names was low; this finding agrees with similar studies [9]. Lack of prescribers’ trust in generic substitutes and presumed therapeutic failure has been shown to influence generic prescribing [21]. The continued low generics prescribing calls for interventions to be put in place to improve prescribing quality. Injection encountered was very high; this is similar to another study carried out among in-patients in Nigeria [9]. Although we did not ascertain the severity of illnesses, it is vital to curtail the use of injections. This will minimize the risk of infection and save cost [1].

Measures should be established to improve the quality of antimicrobial prescription, delivery, and use. Most prescribers understand that overprescription of antibiotics may lead to antibiotic resistance; however, they admit to the overuse of antibiotics and the prescription of antibiotics in the absence of bacterial infection [22]. Antibiotics were the third leading cause of adverse drug reactions reported in the prospective cohort study of adverse drug reaction monitoring on medical wards [13]. This has a consequential potential increase in health care cost and warrants a pragmatic strategy to curbing the menace. A multidisciplinary approach can improve the quality of antibiotics prescription, reducing cost and curbing infection/resistance [23]. Interventions such as setting up antimicrobial stewardship committees, continuing in-service face-to-face medical education as a licensure requirement, and supervision, audit, and feedback systems are effective in promoting the rational use of antibiotics [1]. Antimicrobial stewardship (AMS) programs have been set up in many institutions globally, including the University College Hospital, Ibadan, Nigeria, where this study was conducted. These are a set of interventions to regulate and promote optimal use of antimicrobials for the best clinical outcomes for patients with a goal to reduce the resistance rate by microbes. This was the focus of a recent study seeking to incorporate this into medical students’ training [24]. In South Africa, an antibiotic stewardship program, consisting of online education, a dedicated antibiotic prescription chart, and weekly dedicated ward rounds, was shown to reduce antibiotics consumption four years from implementation [25]; similar measures that are in place in Nigeria may be responsible for the reduction in injection use. Monthly audits of antibiotics prescription quality can also be introduced. A good case study is the monthly level audit dubbed “The Champions League” where a drug card stating the start date, duration of treatment, and indication for antibiotic prescribing is used for every in-patient receiving an antibiotic and compared across subspecialty wards [26].

Our study had some limitations. Data were obtained from only one hospital. However, this large hospital serves a large proportion of people in the southwest of Nigeria. The data were also obtained in 2012. A study on current antimicrobial use is ongoing; it will be interesting to see how much trends in antimicrobial prescription have changed over this period of time. Information on the trend in antimicrobial use will help us design further studies to improve the use of antibiotics in resource-limited settings. Also, we were not able to get information on pathogens isolated from the tests performed on the small proportion of patients.

In this study, we were able to establish the prescribing patterns of antibiotics with emphasis on the DDDs of antibiotics prescribed during our study period.

## Conclusions

There is an unabated prescription of low generics. This calls for interventions to be put in place to forestall irrational prescriptions in order to improve prescribing quality. Parenteral formulation prescription was very high, it is vital to curtail the use of parenteral formulations. This will minimize the risk of infection. However, the use of parenteral formulations may not be unexpected in patients who are admitted to a hospital and are often very sick, requiring prompt treatment with intravenous drugs.

Irrational antibiotics prescription is undoubtedly a serious concern in Nigeria. Drug utilization research using the ATCC/DDD metric should be entrenched in monitoring trends of drug use over time. Improvement of antibiotics stewardship and the rational use of antibiotics will be enhanced.
